# Exploring the ethical implications in the telesurgery ERA

**DOI:** 10.1590/S1677-5538.IBJU.2024.0133

**Published:** 2024-03-10

**Authors:** Marcio Covas Moschovas, Shady Saikali, Travis Rogers, Ahmed Gamal, Roshane Perera, Sumeet Reddy, Vipul Patel

**Affiliations:** 1 AdventHealth Global Robotics Institute Celebration FL USA AdventHealth Global Robotics Institute, Celebration, FL, USA; 2 University of Central Florida Orlando FL USA University of Central Florida (UCF), Orlando, FL, USA

*To the editor*

In the current era of medical innovation with robotic surgery, 3-dimensional (3D) imaging, and high-speed telecommunication, the horizon of surgical procedures expands beyond traditional operative rooms (1, 2). In this scenario, Telesurgery emerges as a humanitarian instrument to improve healthcare delivery worldwide (3). However, as this novel field expands in multiple directions, the ethical implications surrounding Telesurgery demand our vigilant attention.(3, 4) In this brief exploration, after our experience doing several Telesurgery Urological cases in Asia, we described crucial ethical considerations highlighting the dynamic connection between medicine, technology, and remote surgery.

The surgeon-patient relationship is an initial concern regarding Telesurgery because the remarkable potential for remote interventions introduces the challenge of preserving this relationship across physical distances. The inherent absence of direct in-person interactions can reduce the interpersonal relationship, potentially impacting the development of a deep connection with the patient. However, after COVID-19, we witnessed an exponential increase in the use of telemedicine, bringing patients and surgeons into close contact during difficult times.

Dehumanization and Objectification are also ethical concerns because Telesurgery focuses on technical precision and might inadvertently cover the patient's unique needs, experiences, and expectations. The virtuosity of advanced robotic platforms can paradoxically distance the surgeon from the patient's individuality, creating an ethical imperative to ensure that every patient remains an individual, and not a mere surgical subject. However, in the current scenario of high-speed communication, we believe patients and surgeons will still maintain a personal interaction.

Patient Vulnerability also takes some new dimensions in Telesurgery. The remote nature of these procedures introduces challenges in responding to complications and ensuring patient safety, potentially magnifying the ethical importance of well-informed decision-making and comprehensive informed consent. Additionally, the need for Telesurgery highlights the limitations of local surgeons in handling that specific procedure. However, emergency procedures such as cardiovascular and neuro, in which time to treatment is crucial, would decrease mortality, sequels, and disability. In addition, complex oncologic surgery would have better outcomes if a remote expert were able to access the console in critical parts of the procedure.

Informed consent is one of the pillars of patient autonomy in all medical areas. The novel challenges posed by remote surgical interventions requires redefining what represents comprehensive and adequate informed consent. Patients must be empowered to fully understand the procedure, its limitations, potential implications, additional plans, and surgeon relations with companies. In this context, we believe the local and remote surgeons' role should also be detailed in the consent.

As Telesurgery transcends national borders, creating legal and jurisdictional plans is crucial to guarantee patient well-being and ethical foundations (5). Determining responsibility in case of adverse outcomes, licensure across jurisdictions, and the need for international collaboration highlight the ethical need for a harmonized legal agenda that ensures patient safety and professional responsibility. Malpractice obligations for surgeons and hospitals and reimbursement rules are mandatory areas in developing a solid Telesurgery network worldwide.

Finally, cyberattacks and patient confidentiality also should be ethical concerns in Telesurgery. Integrating technology and patient data exposes vulnerabilities that demand vigilance against cyberattacks, ensuring surgical integrity and maintaining patient confidentiality. Previous studies described several types of cyberattacks, such as manipulation of surgeon's actions, delays, or even obstructing movements (6). In this context, Telesurgery providers should invest in cybersecurity with dedicated lines for surgery transmission before starting a program.

In this editorial, we expose some major ethical dilemmas in the field of Telesurgery. As surgeons, society members, and technological innovators, discussing and addressing the above subjects are crucial to establishing a safe Telesurgery routine. Ensuring patient autonomy, transparent communication, and preserving trust in the surgeon-patient relationship are ethical imperatives in all medical fields. In this context, it is vital to ensure that a solid commitment to patient well-being, trust, and ethical responsibility follows Telesurgery's potential for expanded access and improved care.
